# Deep learning neural network development for the classification of bacteriocin sequences produced by lactic acid bacteria

**DOI:** 10.12688/f1000research.154432.2

**Published:** 2025-06-20

**Authors:** Lady L. González, Isaac Arias-Serrano, Fernando Villalba-Meneses, Paulo Navas-Boada, Jonathan Cruz-Varela

**Affiliations:** 1 School of Biological Sciences and Engineering, University Yachay Tech, Urcuqui, Provincia de Imbabura, 100119, Ecuador

**Keywords:** Deep Learning Neural Network, Bacteriocin, Lactic Acid Bacteria, K-mers, Embedding Vectors

## Abstract

**Background:**

The rise of antibiotic-resistant bacteria presents a pressing need for exploring new natural compounds with innovative mechanisms to replace existing antibiotics. Bacteriocins offer promising alternatives for developing therapeutic and preventive strategies in livestock, aquaculture, and human health. Specifically, those produced by LAB are recognized as GRAS and QPS. This study aims to develop a deep learning model specifically designed to classify bacteriocins by their LAB origin, using interpretable k-mer features and embedding vectors to enable applications in antimicrobial discover.

**Methods:**

We developed a deep learning neural network for binary classification of bacteriocin amino acid sequences (BacLAB vs. Non-BacLAB). Features were extracted using k-mers (k=3,5,7,15,20) and vector embeddings (EV). Ten feature combinations were tested (e.g., EV, EV+5-mers+7-mers). Sequences were filtered by length (50–2000 AA) to ensure uniformity, and class balance was maintained (24,964 BacLAB vs. 25,000 Non-BacLAB). The model was trained on Google Colab, demonstrating computational accessibility without specialized hardware.

**Results:**

The ‘5-mers+7-mers+EV’ group achieved the best performance, with k-fold cross-validation (k=30) showing: 9.90% loss, 90.14% accuracy, 90.30% precision, 90.10% recall and F1 score. Folder 22 stood out with 8.50% loss, 91.47% accuracy, and 91.00% precision, recall, and F1 score. Five sets of 100 LAB-specific k-mers were identified, revealing conserved motifs. Despite high accuracy, sequence length variation (50–2000 AA) may bias k-mer representation, favoring longer sequences. Additionally, experimental validation is required to confirm the biological activity of predicted bacteriocins. These aspects highlight directions for future research.

**Conclusions:**

The model developed in this study achieved consistent results with those seen in the reviewed literature. It outperformed some studies by 3-10%. Its implementation in resource-limited settings is feasible via cloud platforms like Google Colab. The identified k-mers could guide the design of synthetic antimicrobials, pending further in vitro validation.

## Introduction

The emergence of antibiotic-resistant bacteria and the rise of new diseases are critical challenges that demand the search for new natural compounds with innovative mechanisms of action to support or replace current antibiotics in use. ^1^^,^^2^ Some bacteria have the ability to produce antimicrobial proteins to inhibit or kill other nearby bacteria. This serves as a form of microbial competition and defense. ^3^ These antimicrobial proteins, known as bacteriocins, are effective against related or similar bacteria to those that produce them, but generally do not affect other organisms such as human or animal cells. ^4^^,^^5^ Bacteriocins have emerged as alternatives for treating urinary tract, skin, respiratory, gastrointestinal infections, among others. They provide additional or alternative treatment options compared to conventional antibiotics. ^6^^–^^8^

A summary of the classification of bacteriocins can be seen in Table 1.

**Table 1.** Classification and characteristics of bacteriocins. This table summarizes the different classes of bacteriocins, detailing their molecular mass, properties, structural characteristics, and examples.

Classification		Characteristics	Examples	Reference
**Class I (lantibiotics)**	Subclass Ia Subclass Ib	**Molecular mass**: *<*5 kDa. **Properties:** resistant to proteolysis, thermostable, and resistant to pH. **Structure:** intramolecular cyclic, providing rigidity and resistance to the action of proteases.	Nisin, Subtilin, Mersacidin	^9^^–^^13^
**Class II (non- antibiotics)**	Subclass IIa Subclass IIb Subclass IIc Subclass IId	**Molecular mass**: *<*10 kDa. **Properties**: thermostable, pH resistant, and ability to depolarize bacterial cell membranes. **Structure**: amphipathic helical with disulfide bridges that increase the stability of the peptide.	Pediciona, Plantaricin, Lactococcin A	^9^^–^^12^^,^^14^
**Class III**	Subclass IIIa Subclass IIIb	**Molecular mass**: *>*30 kDa. **Properties**: thermolabile, and unmodified. They have two mechanisms of action: lytic and non-lytic. **Structure**: large proteins.	Helviticin J, Millericin B	^9^^,^^10^^,^^14^
**Class IV**	-	**Molecular mass**: - **Properties**: thermostable, and resistant to pH. **Structure**: large peptides with complex structure.	Lactocin S, Eenterocin AS-48, Circularin	^10^

A common type of bacteria known to produce bacteriocins is Lactic Acid Bacteria (LAB). ^15^ Additionally, LABs are particularly intriguing due to the long history of safe use of some strains and their status as “Generally Recognized as Safe” (GRAS), along with the “Qualified Presumption of Safety” (QPS) that most LAB strains possess. ^16^^,^^17^ Typically, LABs are either cocci or rods and encompass over 60 genera. The major genera include *Aerococcus, Carnobacterium, Enterococcus, Lactobacillus, Lactococcus, Leuconostoc, Oenococcus, Pediococcus, Streptococcus, Tetragenococcus, Vagococcus, Propionibacterium, Bifidobacterium, and Weisella.* ^2^^,^^18^

Although these genera include the main producers of bacteriocins, ^15^ their uneven representation in public databases may introduce bias. For example, in UniProt, the genus *Lactobacillus* accounts for over 60% of LAB bacteriocin sequences, ^19^ while genera such as *Weissella* or *Vagococcus* are underrepresented (less than 5% each). To mitigate this risk, our study: employs stratified cross-validation that preserves taxonomic proportions, and includes sequences from all selected genera, even the less common ones (see Methods). However, we acknowledge that the full diversity of bacteriocin-producing LAB is still not captured in the available databases.

Bacteriocins produced by LAB have gained popularity due to their promising applications in the food industry as natural preservatives. This reduces the need for adding chemical preservatives or applying physical treatments during food production. ^20^^,^^21^ Additionally, they can be used within the pharmaceutical and medical industry, serving as therapeutic agents or alternatives to traditional antibiotics. ^22^ Bacteriocins derived from LABs are colorless, tasteless, and odorless. Moreover, they possess several crucial metabolic traits such as strong tolerance to low pH, the ability to produce acid and aroma, protein hydrolysis, production of viscous exopolysaccharides, and resilience to high thermal stress. ^12^^,^^23^^,^^24^

On the other hand, the development of machine learning and artificial intelligence techniques, coupled with the availability of sequenced bacterial genomes, has enabled the use of new techniques in bioinformatics. In the context of bacteriocins, employing neural networks allows for the identification of patterns in amino acid sequences (aa), providing an advantage in discovering new bacteriocins that remain uncharacterized. ^25^^,^^26^ This research is based on the need to efficiently identify bacteriocin sequences produced by LAB, ^27^^,^^28^ as the genetic and structural diversity of these peptides poses a challenge. ^29^ Therefore, a deep learning neural network was developed for the binary classification of bacteriocin amino acid sequences, distinguishing between those produced by lactic acid bacteria (BacLAB) and non-BacLAB. Feature extraction using the k-mer method and vector embedding was employed.

## Fields where bacteriocins can be applied to address diverse issues

### Food industry

Some microorganisms can cause food and beverage contamination, leading to their deterioration, posing a constant concern in the food industry as it can spoil taste and cause foodborne illnesses in humans. ^30^^,^^31^ Bacterial pathogens transmitted through food are the primary cause of food poisoning. Chemical additives have been widely used for food preservation; however, their toxicity may raise human health issues. Some of the commercially used chemical preservatives include various synthetic chemicals. ^32^^,^^33^ Currently, there is a negative public perception towards chemical preservatives. This has led to a consumer preference for alternatives considered more “natural”. ^34^

In response to this demand for natural preservatives, bacteriocins show significant potential for use in the food industry, aiming to prevent food spoilage and hinder disease transmission by inhibiting the growth of pathogenic bacteria. ^34^^,^^35^ Certain LAB-derived bacteriocins, such as nisin, pediocin, enterocin, and leucocin, have been employed for this purpose. ^36^^–^^38^ They can be used in the preservation of dairy products, meats, vegetables, sourdough bread, wine, among others. ^2^ Furthermore, using bacteriocins as preservatives leads to the creation of tastier, less acidic, lower salt content, and higher nutritional value food products. Additionally, these bacteriocins can be used as antimicrobial films in food packaging to extend the shelf life and expiration dates of these products. ^39^^,^^40^

However, it’s important to note that while bacteriocins are a promising tool, their application is still under development and study, and they do not completely replace traditional antibiotics in all cases. Further research is needed to fully understand their potential and limitations. ^34^

### Medicine

Currently, the growing resistance of bacterial pathogens poses a serious challenge to global public health, impacting not only humans but also animals, plants, and the environmental ecosystem. ^41^ Drug resistance is on the rise worldwide due to the excessive and uncontrolled use of antimicrobial substances. According to the WHO, superbugs represent one of the most significant threats to public health, causing millions of deaths each year. ^42^ It is projected that by 2060, at least 20 new types of antibiotics will be needed to effectively address the problem of bacterial drug resistance. However, developing new antibiotics involves a long and complex process, posing a significant barrier. Therefore, it is imperative to explore and develop new therapeutic strategies capable of effectively combating antibiotic-resistant microorganisms. ^7^^,^^18^

In clinical applications, some bacteriocins have demonstrated efficacy in treating infections, especially those caused by multidrug-resistant strains. Being produced by non-pathogenic bacteria that typically colonize the human body, they are of interest in the medical field. ^43^^–^^45^ Some identified bacteriocins applicable in the treatment of infectious diseases include nisin, lacticin, salivaricin, subtilosin, mersacidin, enterocin, gallidermin, epidermin, and fermentin. ^30^ Furthermore, bacteriocins have been explored for potential use in treating conditions such as diarrhea, dental caries, mastitis, and cancer. ^46^^–^^48^

### Livestock animal husbandry

Livestock, comprising domestic animals raised in agricultural settings, play a crucial role in providing labor and a wide range of products such as milk, meat, eggs, hides, and leather. Maintaining livestock health and improving the economy through optimal production requires proper feeding and effective hygiene practices. However, farm animals remain susceptible to infections caused by viruses and bacteria despite these measures. ^49^^–^^51^

In the quest to safeguard animal health on farms, novel techniques are being explored as alternatives to antibiotics. This search becomes especially relevant due to various infectious diseases caused by bacteria in cattle, including conditions like mastitis, post-weaning diarrhea, meningitis, arthritis, endocarditis, pneumonia, and septicemia. Despite this pressing need, the range of bacteriocins evaluated for maintaining livestock health is limited, primarily focusing on nisin, lacticin, garvicin, and macedocin. ^52^^–^^54^

The application of bacteriocins in livestock food or water has ensured food safety by reducing the presence of foodborne pathogens in the gastrointestinal tract. ^55^^,^^56^ This application of bacteriocins has not only been used to improve the productivity of cattle but also probiotic strains capable of producing bacteriocins have been explored to increase the growth rate of pigs. Furthermore, efforts have been made in the poultry industry to control Salmonella. ^57^ Maintaining a diet with bacteriocin-producing bacteria can reduce existing populations of foodborne pathogens such as Salmonella and Escherichia coli and prevent the reintroduction of these pathogenic bacteria. ^55^ Additionally, they can be used in other forms such as the development of intra-mammary formulations for mastitis, which act as germicidal preparations applied to cows’ udders. ^58^^,^^59^

### Aquaculture

Aquatic cultures face similar challenges to livestock, dealing with potential pathogenic risks and requiring preventive measures such as various breeding techniques, vaccination, and antibiotic use. ^55^^,^^60^ Bacteriocins function as probiotics, leveraging the interconnected ecosystem shared by animals and microorganisms within the aquatic environment. This interaction promotes probiotic competition against pathogenic bacteria, facilitating the production of inhibitory compounds. As a result, it improves water quality, strengthens the immune response of host species, and enhances species nutrition by producing additional digestive enzymes. ^61^^–^^63^

Studies involving photosynthetic bacteria like Rhodobacter sphaeroides and bacteriocins derived from Bacillus spp. have investigated their impact as probiotics on shrimp growth and digestive enzyme activity. ^64^^,^^65^ Likewise, experiments with nutrient-enriched water using Alchem Poseidon, a blend of *Bacillus subtilis*, *L. acidophilus, Clostridium butyricum, and Saccharomyces cerevisiae*, have shown potential for preventing infections, as the administered bacteria successfully colonized both the host and the aquatic environment. ^66^^,^^67^

### Work related to artificial intelligence for the classification of bacteriocin sequences

Among the works carried out using deep learning neural networks to analyze large datasets and achieve accurate classification of bacteriocins is the article by Poorinmohammad et al. (2018). ^68^ In this study, peptide sequence analysis is conducted using machine learning alongside feature selection, and a Sequential Minimal Optimization (SMO)-based classifier is developed to predict lantibiotics, achieving precision and specificity values of 88.5% and 94%, respectively. However, this approach was limited to lantibiotics (Class I bacteriocins) and did not address the structural diversity of other bacteriocin classes.

Furthermore, in the work of Yount et al. (2020), ^69^ the BACII algorithm was created to identify and classify bacteriocin sequences. This algorithm integrates a consensus signature sequence, physicochemical elements, and genomic patterns within a high-dimensional query tool to select peptides resembling bacteriocins. It accurately retrieved and distinguished almost all known class II bacteriocin families, achieving a specificity of 86%. While innovative, BACII’s reliance on predefined class II motifs limits its applicability to novel or atypical bacteriocin families. In the article by Akhter and Miller (2022), a similar approach was taken, where a machine learning-based software tool was developed to extract potential features from bacteriocin and non-bacteriocin sequences, considering their physicochemical and structural properties. Support Vector Machine (SVM) and Random Forest (RF) algorithms were employed. In this article, a precision of 95.54% was achieved. ^70^ Notably, this tool used small datasets (<1,000 sequences), which may restrict its generalization to broader bacteriocin diversity.

Various methods have also been used to identify bacteriocins from bacterial genomes based on bacteriocin precursor genes or contextual genes. For instance, BAGEL ^71^ and BACTIBASE ^72^ are online tools that analyze experimentally validated and annotated bacteriocins, similar to the BLASTP protein search tool. These tools rely on methods that facilitate the identification of potential bacteriocin sequences based on the homogeneity of known bacteriocins. However, these similarity-based approaches suffer from two critical limitations. They inherently exclude bacteriocins with low homology to known sequences, and their databases are biased toward well-studied LAB genera (e.g., Lactobacillus), underrepresenting rare producers like Weissella or Vagococcus. This issue led to the development of the BOA software, ^73^ which attempts to address this problem by integrating prediction tools based on the conservation of contextual genes from the bacteriocin operon. Nevertheless, they still rely on genomic searches based on homology.

There are taxonomic bias in existing tools. A recurring challenge in bacteriocin prediction is the overrepresentation of certain LAB genera (e.g., Lactobacillus, Enterococcus) in public databases, which may skew models toward recognizing features specific to these groups. For example, in UniProt, >60% of annotated bacteriocin sequences derive from just three genera, potentially marginalizing structurally unique peptides from less-studied LAB. This bias could lead to false negatives in ecological or industrial applications where microbial diversity is crucial.

In addition, the study by Nguyen et al. (2019) utilized a different technique from the previous methods by applying word embeddings of protein sequences to represent bacteriocins. This approach takes into account the amino acid order in protein sequences to predict new bacteriocins from sequences without relying on sequence similarity. While promising for novel bacteriocin discovery, their model was trained on limited data and did not account for taxonomic imbalances in sequence sources. This method even enables the prediction of potentially unknown bacteriocins with high probability. Overall, representing sequences with word embeddings that preserve information about the sequence order can be applied to peptide and protein classification problems where sequence similarity cannot be used. ^74^

Similarly, in the work by Hamid and Friedberg (2019), ^75^ word embedding was used to identify bacteriocins, representing protein sequences using Word2vec. These representations were used as inputs for various deep recurrent neural networks (RNNs) to distinguish between bacteriocin and non-bacteriocin sequences. This technique addresses challenges such as diversity among bacteriocin sequences. Though effective, their RNN architecture required manual tuning for different bacteriocin classes, reducing scalability. Meanwhile, Fields et al. (2020) developed a process for designing and testing bacteriocin-derived compounds. They employed machine learning and a filter of biophysical features to generate an algorithm that predicts bacteriocins. This involved generating characteristic sequences of 20-mers. ^26^ A key limitation was their focus on short peptides (≤50 AA), excluding larger bacteriocins like Class III.

Current bacteriocin prediction tools, such as BAGEL ^71^ and BACII, ^69^ fail to address two critical needs in the field. First, they lack taxonomic resolution, making them unable to distinguish bacteriocins produced by lactic acid bacteria (LAB) from those synthesized by other bacterial groups. Second, they depend heavily on sequence homology, which limits their ability to detect structurally novel bacteriocins, particularly those originating from understudied LAB genera. This gap significantly restricts their usefulness in industrial and therapeutic contexts, where the specific taxonomy of the bacteriocin-producing organism is essential for applications such as probiotic development, targeted antimicrobial design, and food safety strategies.

To overcome these limitations, our study uses a balanced dataset spanning all major bacteriocin classes and LAB genera (without genus-specific evaluation), employs k-mer features independent of sequence homology, and validates performance on a generalized LAB group to ensure broad applicability (see Methods).

Additionally, there are other works that use antimicrobial peptide (AMP) sequences. However, it’s important to note that all bacteriocins are antimicrobial peptides, but not all antimicrobial peptides are bacteriocins. For example, in the study by Li et al. (2022), ^76^ they present a deep learning model called AMPlify for antimicrobial peptide prediction. The cross-validation results for the model achieve 91.70% accuracy, 91.40% sensitivity, 92.00% specificity, and 91.68% F1 score.

Similarly, in Wang et al. (2023), ^77^ they developed a bidirectional short and long-term memory deep learning network called AMP-EBiLSTM with an accuracy of 92.39%. This approach employs a binary profile function and a pseudo-amino acid composition to capture local sequences and extract amino acid information. In another study, a model known as AMP-BERT was developed. This network uses a bidirectional transformer encoder (BERT) architecture to extract structural and functional information from input peptides, categorizing each input as AMP or non-AMP. Notably, this network achieved a correct prediction rate of 76% for external test sequences selected in this research. ^78^

Similarly, a system called AMPs-Net was introduced, an algorithm designed to streamline experimentation and improve the efficiency of discovering potent AMPs. It exhibited good prediction of the antibacterial capabilities of numerous peptides, with an average accuracy ranging from 80.98% to 91.2% and precision varying from 75.77% to 94.26%. ^79^ In the study by Gull et al. (2019), they achieved 97% accuracy for an algorithm that identifies biologically active and antimicrobial peptides. ^80^ Similarly, in the study by Redshaw et al. (2023), a neural network was developed to predict the antimicrobial activity of sequences. It was trained on two different databases, achieving a precision result of 86-92% for one database and 72-77% for the other. ^81^

In another work, an application used for predicting antimicrobial peptides based on properties achieved an accuracy exceeding 80% and sensitivity above 90%. ^82^ In the study by Yan et al. (2020), a method for predicting short-length antimicrobial peptides (≤ 30 aa) is presented. Their convolutional neural network, called Deep-AmPEP30, demonstrated a 77% accuracy rate. ^83^ Additionally, in the study by Veltri et al. (2018), a deep learning neural network using embedding vectors to reduce weights when processing sequences was developed. It was shown that antimicrobial peptides could be constructed using only nine amino acids, achieved through the k-mers method. The network achieved an accuracy of 90.55%. ^84^

The primary aim of this study is to develop a deep learning model that accurately distinguishes bacteriocin sequences produced by lactic acid bacteria (LAB) from non-LAB bacteriocins. Unlike existing tools that classify bacteriocins generically, our approach specifically targets the LAB/non-LAB dichotomy, enabling applications in probiotic development and food safety. It uses k-mer signatures and embedding vectors to overcome the limitations of homology-based methods and provides interpretable features (100 characteristic k-mers per length) to guide synthetic peptide design.

## Methods

**Figure 1.**
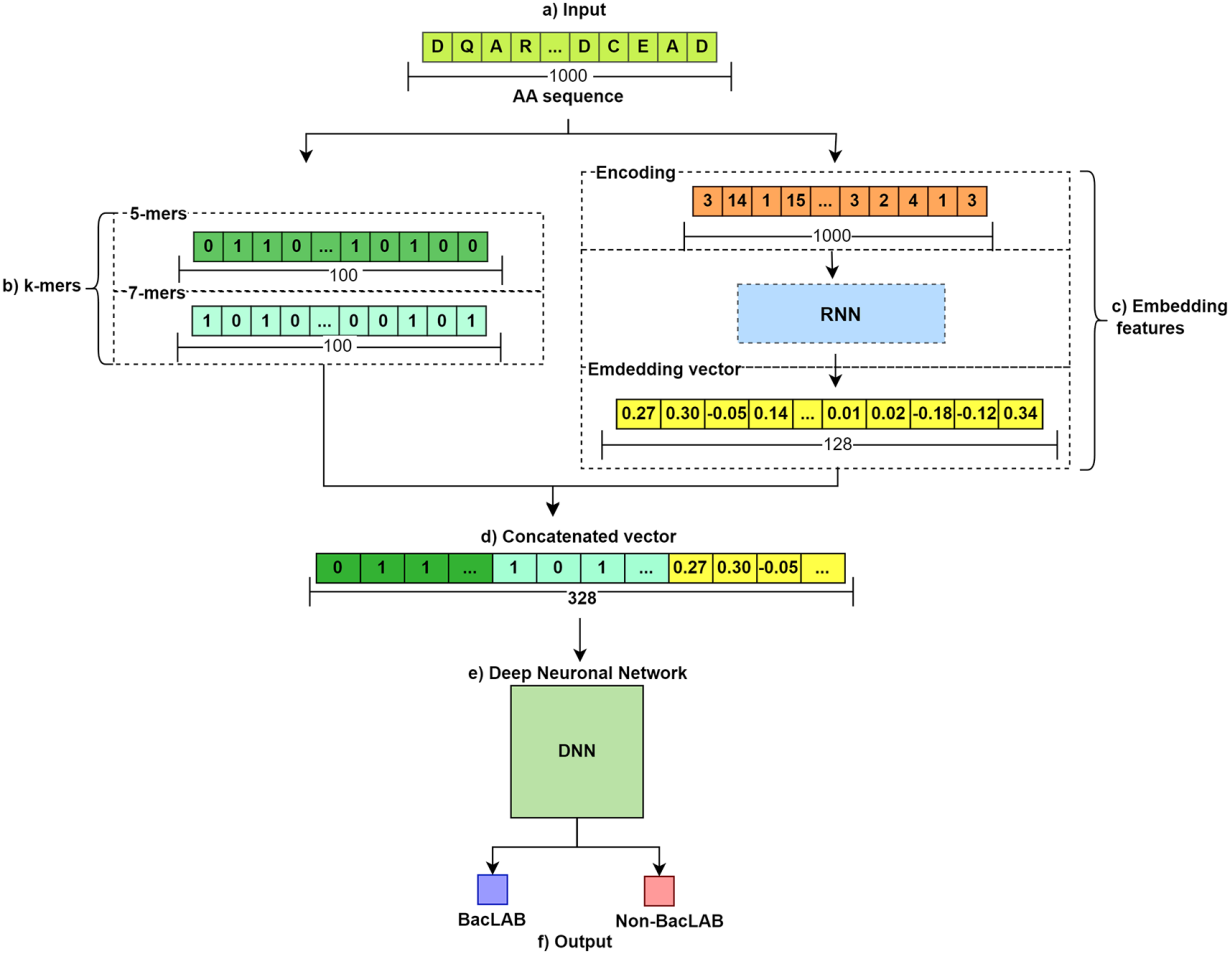
Methodological workflow for predicting bacteriocin AA sequences. This figure illustrates the comprehensive flow of the method used to predict bacteriocin amino acid sequences in BacLAB and Non-BacLAB groups.

The general flow of the method used is illustrated in Figure 1. In section a), the input of the AA sequences is shown. There are two groups: BacLAB and Non-BacLAB. Subsequently, feature extraction is performed for each sequence. Two methods were employed. In b), the use of k-mers to obtain vectors of 0s and 1s representing the presence or absence of representative k-mer groups is shown. The resulting vectors have a length of 100. Meanwhile, in c), a 128-character embedded vector is obtained by passing the sequence through an RNN. These features are concatenated in d). The resulting concatenation serves as input for the DNN in step e). Finally, in f ), a prediction of the aa sequences entered into the trained model is made. Training and validation were performed on Google Colab (a cloud-based environment with free GPUs), confirming that the model is computationally efficient and replicable without investment in expensive infrastructure.

### Data collection

The AA sequences from both BacLAB and Non-BacLAB were obtained using the publicly accessible UniProt database, downloaded in xlsx format using the Excel option on the platform. ^19^ The search on this platform was conducted using the keyword “bacteriocin.” The retrieved parameters for each bacteriocin include: Entry, Organism, Length, and Sequence. Additionally, considering the binary classification, a column was added to label the sequences. The BacLAB dataset was labeled as 1, while the Non-BacLAB sequences were labeled as 0.

To classify which sequences correspond to BacLAB and which ones to Non-BacLAB, the parameter “organism” was considered to identify the species that produce the bacteriocin. The LAB genera included for classification encompassed *Lactobacillus, Lactococcus, Leuconostoc, Pediococcus, Streptococcus, Aerococcus, Alloiococcus, Carnobacterium, Dolosigranulum, Enterococcus, Oenococcus, Tetragenococcus, Vagococcus, and Weissella.* ^85^

**Figure 2.**
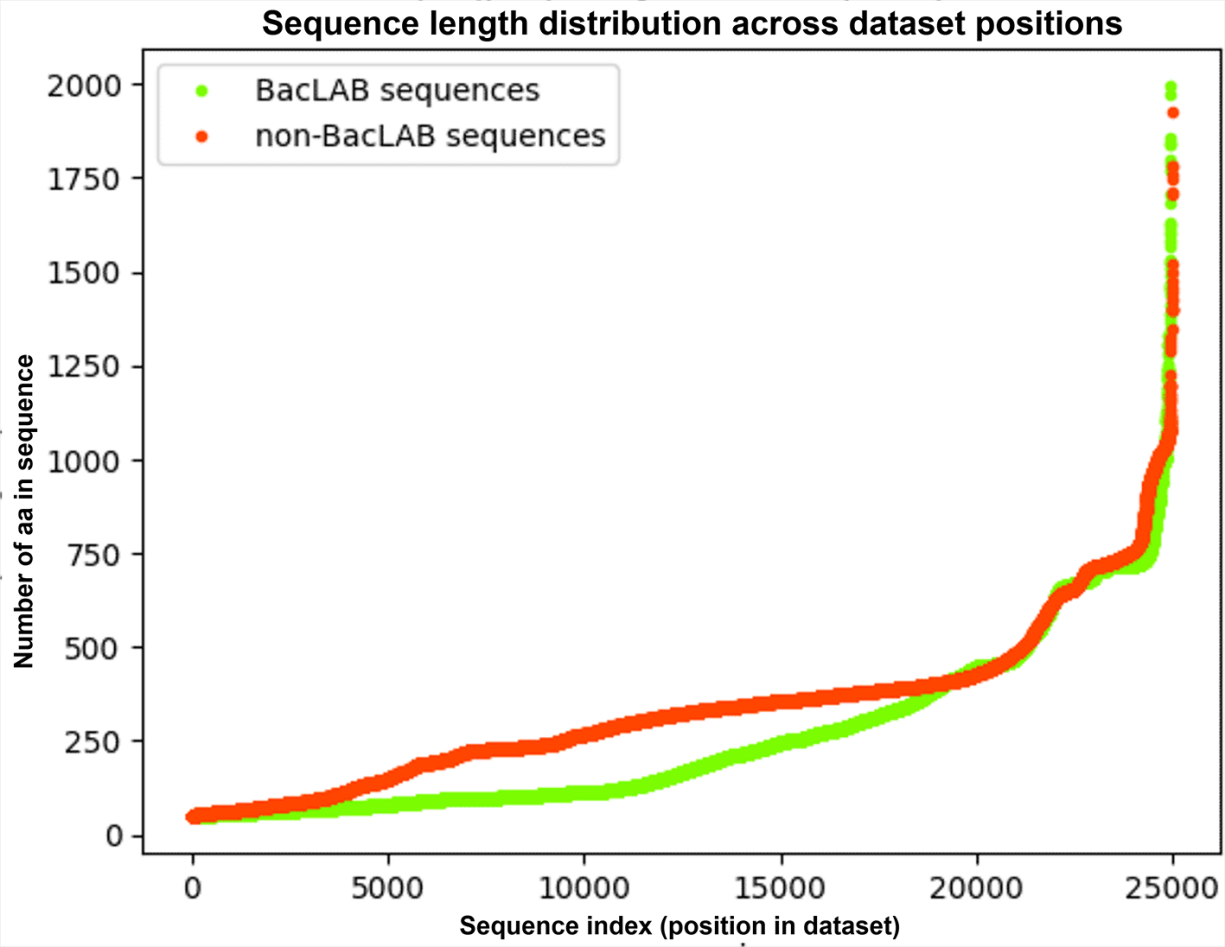
Length of each sequence (BacLAB vs. Non-BacLAB) ordered by dataset position. The curves display the length (in amino acids) of each BacLAB and Non-BacLAB sequence, plotted according to their original position in the dataset. The x-axis represents the sequence index (1 to 25,000), and the y-axis shows the corresponding sequence length. Sequences were filtered to retain lengths between 50 and 2000 amino acids.

Sequences with lengths between 50 and 2000 amino acids were selected to ensure consistency. After filtering, the BacLAB dataset contained 24,964 sequences. For the Non-BacLAB dataset, which originally had a larger number of sequences, a random subset of 25,000 sequences was selected to prevent class imbalance in subsequent analyses. Figure 2 illustrates the length of each individual sequence (y-axis) plotted against its position in the ordered dataset (x-axis), allowing a direct comparison of length trends between BacLAB and Non-BacLAB sequences.

### Feature extraction

K-mers

**Figure 3.**
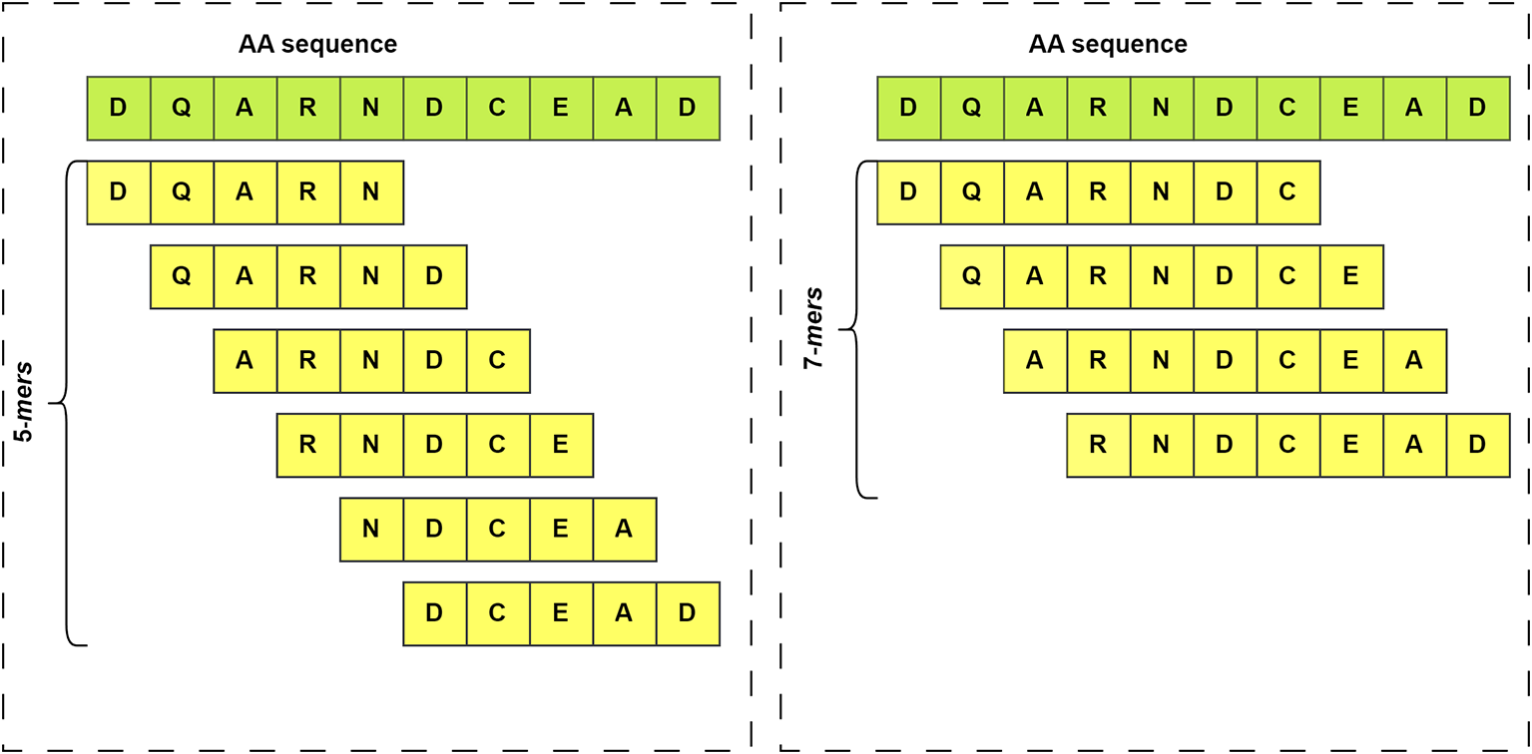
Illustration of k-mers generated from an amino acid sequence with different k-values. On the left side are shown the k-mers that would be obtained from a sequence if k=5 is set. On the right side the same sequence is used, but in this case k=7.

In the realm of amino acid sequence processing (or biological sequences in general) using neural networks, a ‘k-mer’ refers to subsequences of length ‘k’. ^86^ These subsequences are formed by dividing a longer sequence into specific-sized fragments, where ‘k’ represents the size of each fragment. ^87^ For example, a k-mer of size 5 would involve splitting the sequence into all possible subsequences of length 5, as illustrated in Figure 3. The k-mer features of a set of sequences enable the discovery of hidden patterns within that sequence population. Additionally, k-mers are useful for representing sequences in a more manageable way. ^88^

At this stage, a list of the 100 most common k-mers within the BacLAB data set was generated. For this, several values of k were selected (k=3, 5, 7, 15, and 20). The k-mers of each BacLAB sequence were generated. Once all the k-mers were obtained, the frequency of each of them was counted. The 100 k-mers with the highest frequency were selected; this was done for each value of k, resulting in five different lists.

**Figure 4.**
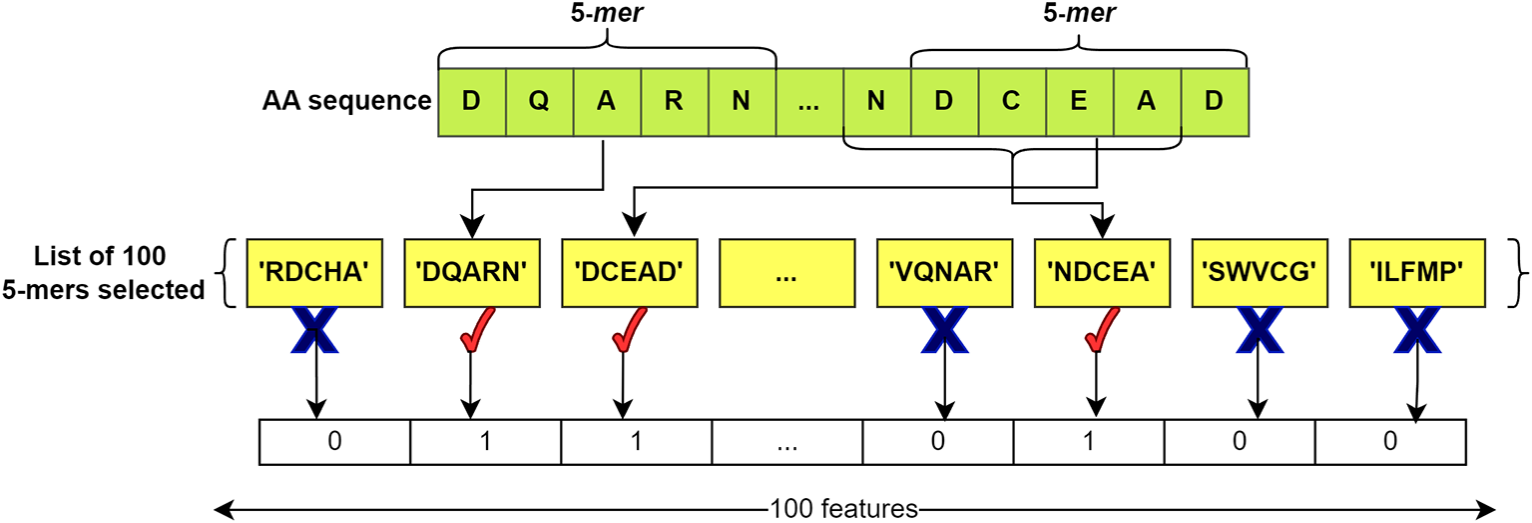
Feature extraction from an AA sequence. The list of 100 selected k-mers is compared with the k-mers of the input sequence. If one of the k-mers of the sequence is found in the list, '1' is added; if it is not found, a '0' is added. This process generates a representative vector for the sequence with 100 features in length. In this example, k=5 is used.

After compiling the lists, feature vectors of ‘0’ and ‘1’ were extracted for each sequence, both for those in the BacLAB and Non-BacLAB groups. The k-mers obtained from each sequence were compared with the list of k-mers. A ‘1’ was assigned if the listed k-mer was present in the analyzed sequence, while a ‘0’ was assigned if the k-mer was not found. This process produced a vector of length 100. Figure 4 illustrates the process.

### Embedding vectors

Word embeddings are numerical representations of amino acids, where each letter denoting an amino acid receives a unique and discrete value. ^89^ Each protein is treated as a distinct input token, and the set of 20 amino acids forms a specific dictionary.

**Figure 5.**
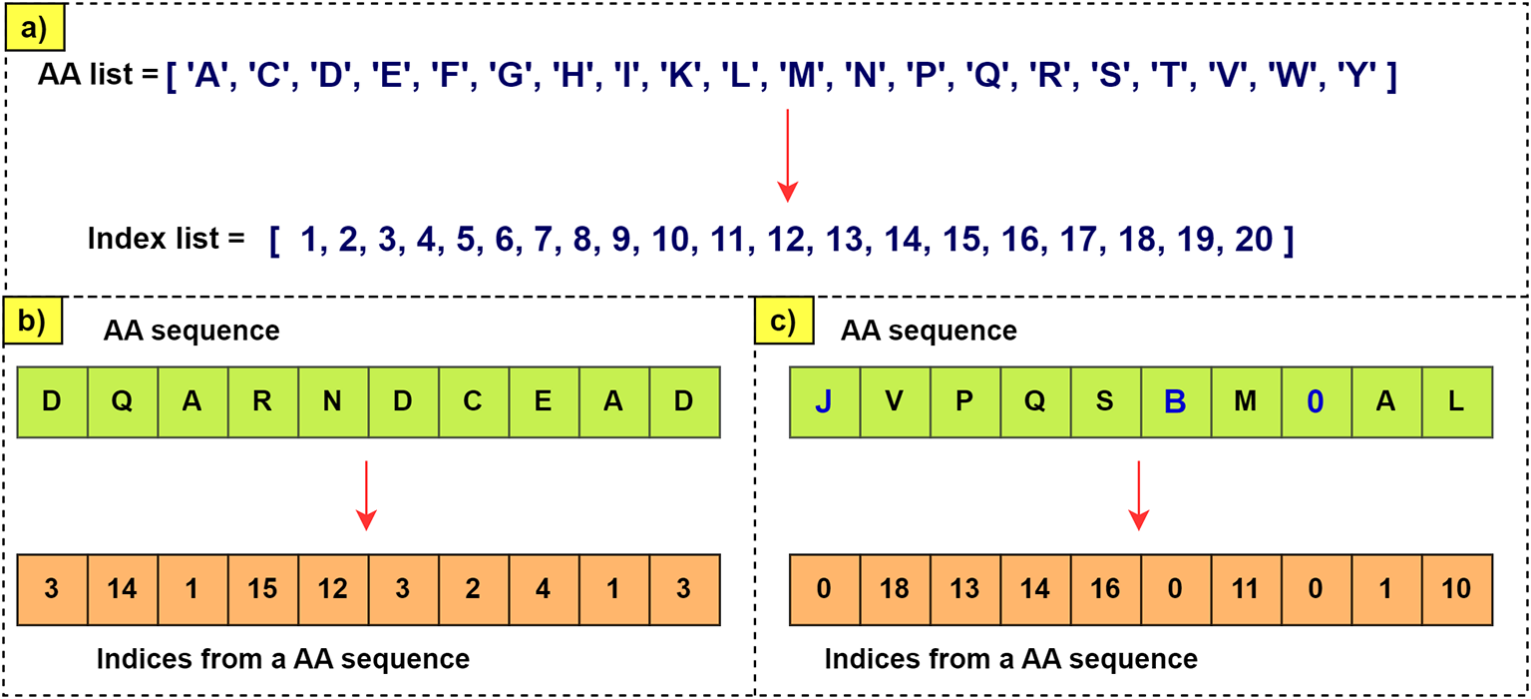
Encoding of amino acid sequences. a) The index number corresponding to each aa is assigned. b) Shows how the sequence is encoded with the indices that correspond to each AA. c) Given that there can be letters or numbers in the sequence that do not exist in the aa list, a value of 0 is assigned as an index. This way, errors are avoided when processing the sequence.

For example, for ‘A’ (Alanine), the index 1 is assigned. Consequently, in a sequence, each occurrence of ‘A’ is denoted with the value 1. Figure 5 clarifies the process of generating the index vector. If letters were to appear in the sequence that are not found in the list of amino acids, they will be represented as zero. These indices are used to encode sequences before introducing them into the neural network that generates the embedded vectors.

Once the index-encoded vectors are obtained, the embedding vectors are extracted. To derive these features, a recurrent neural network (RNN) is applied using the Gated Recurrent Unit (GRU) cell. RNNs with GRUs can handle sequences of varying lengths due to their inherent sequential processing nature and the specific architecture of GRUs. This makes GRU-based RNNs particularly useful in applications where sequence lengths are variable, as they can efficiently handle input length variability without losing learning capacity. ^90^^–^^92^

**Figure 6.**
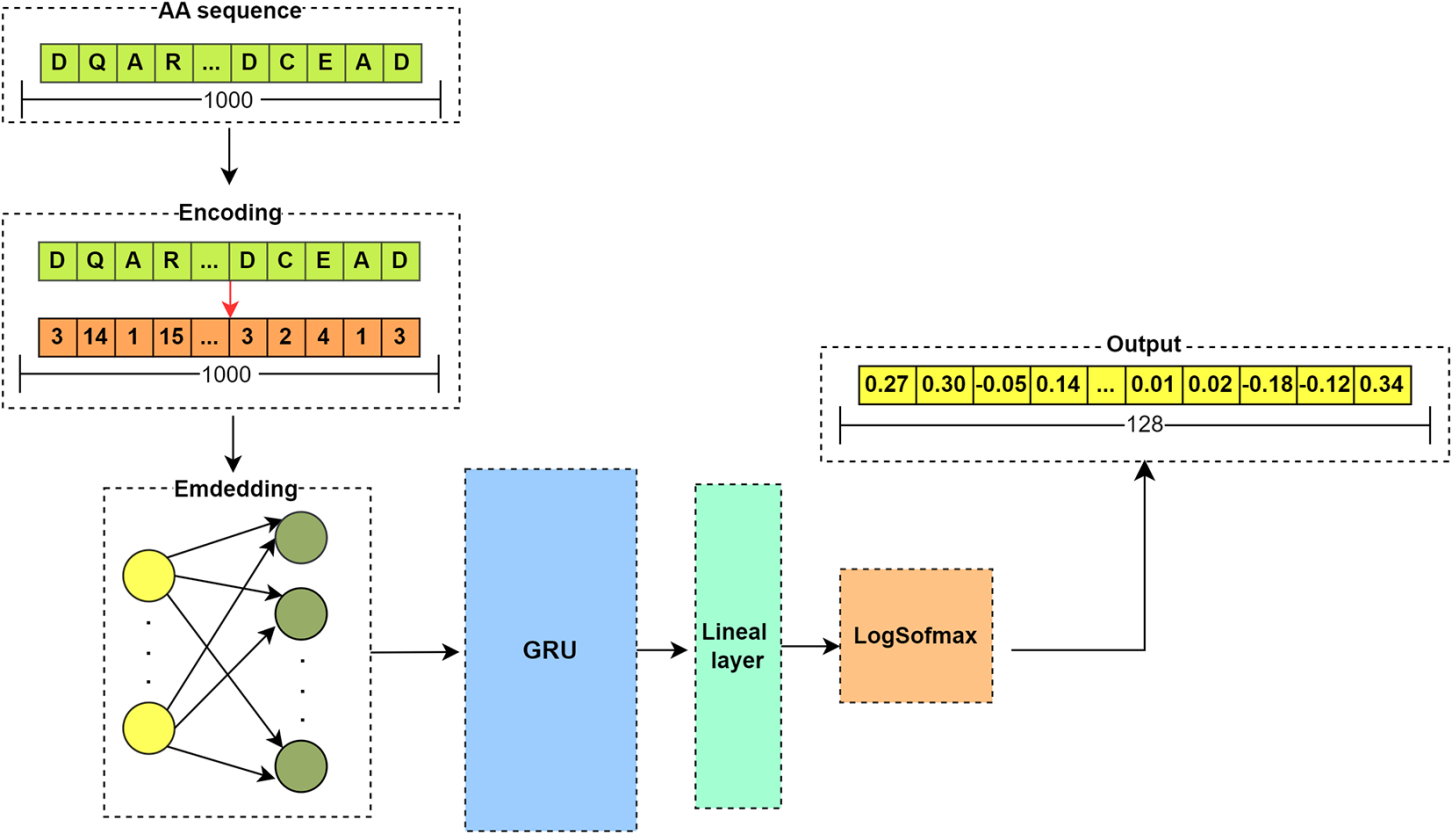
Flowchart of RNN model using GRU cell. Input: Amino acid sequence (top) and its integer encoding (middle). Embedding Layer: Converts encoded indices into dense vectors (128-D) via a pre-trained weight matrix. GRU Layer: Processes sequential data (arrows indicate flow direction), capturing contextual relationships between amino acids. Linear Layer: Final transformation (LogSoftmax) for classification.

The embedding layer in the network acts as a lookup table or a weight matrix where each row represents, in our case, a vectorized representation of a specific amino acid. ^93^ The number of rows is equal to the count of unique elements in the vocabulary, which is the number of amino acids plus one, including index zero reserved for a non-existent variable in the amino acid list. The number of columns represents the embedding dimension, a model hyperparameter set to 128 in this case. Consequently, the length of the embedding vector obtained is also 128 for each sequence. Normally, before training begins, the weight matrix is initialized randomly along with all the network parameters. However, for this step, a pre-trained network is used, loading the weights into the model. Figure 6 illustrates the structure of the RNN model.

### Concatenated data sets

Different datasets will be used to train the neural network and determine which combination of parameters produces the best results. For the selection of k-mers, values of k=3, k=5, k=7, k=15, and k=20 will be used, as shown in Table 2.

**Table 2.** Parameters for neural network training. This table presents the different k-mer values used for training the neural network.

Concatenation groups
EV
EV + 3-mers
EV + 5-mers
EV + 7-mers
EV + 15-mers
EV + 20-mers
EV + 3-mers + 5-mers
EV + 3-mers + 7-mers
EV + 5-mers + 7-mers
EV + 15-mers + 20-mers

These specific values for k (k = 3, 5, 7, 15, 20) were chosen to align with conserved motifs reported in bacteriocin literature, ensuring coverage of both short functional domains and longer structural regions:

• **k = 3–7**: These values target small but critical motifs, such as the 5-AA sequences YGNGV/YDNGI in class IIa bacteriocins, and extended variants (e.g., 7-AA YGNGVXC) associated with antimicrobial activity ^13^^,^^94^^–^^96^
• **k = 15–20**: Longer k-mers were selected to encapsulate the “pediocin box” (e.g., YGNGVXCXXXXCXV, 14 AA; or YGNGVXCXXXXCXVXWXXA, 19 AA), a hallmark of bacteriocin tertiary structure and functionality. ^97^^–^^99^ This range also accommodates similarities in the N-terminal half of sequences (17–19 AA) linked to target specificity. ^98^

By incorporating this spectrum of k-values, our approach balances granularity (capturing short motifs) with context (preserving structural dependencies), a strategy validated in prior studies on peptide classification. ^98^^,^^99^

### Deep neural network

To predict amino acid sequences, a Deep Neural Network (DNN) was employed following the structure described in Jeff et al.’s article. ^99^ This type of network was chosen for its ability to learn complex patterns and representations from data. Additionally, they can efficiently handle large datasets. ^100^ The construction of this neural network used Python 3.10.12 in Google Colab along with several libraries: i) Pandas (RRID:SCR 018214), ^101^ ii) Keras, iii) Scikit-learn (RRID:SCR 002577), ^102^ iv) NumPy (RRID:SCR 008633), ^103^ and v) Matplotlib.

**Figure 7.**
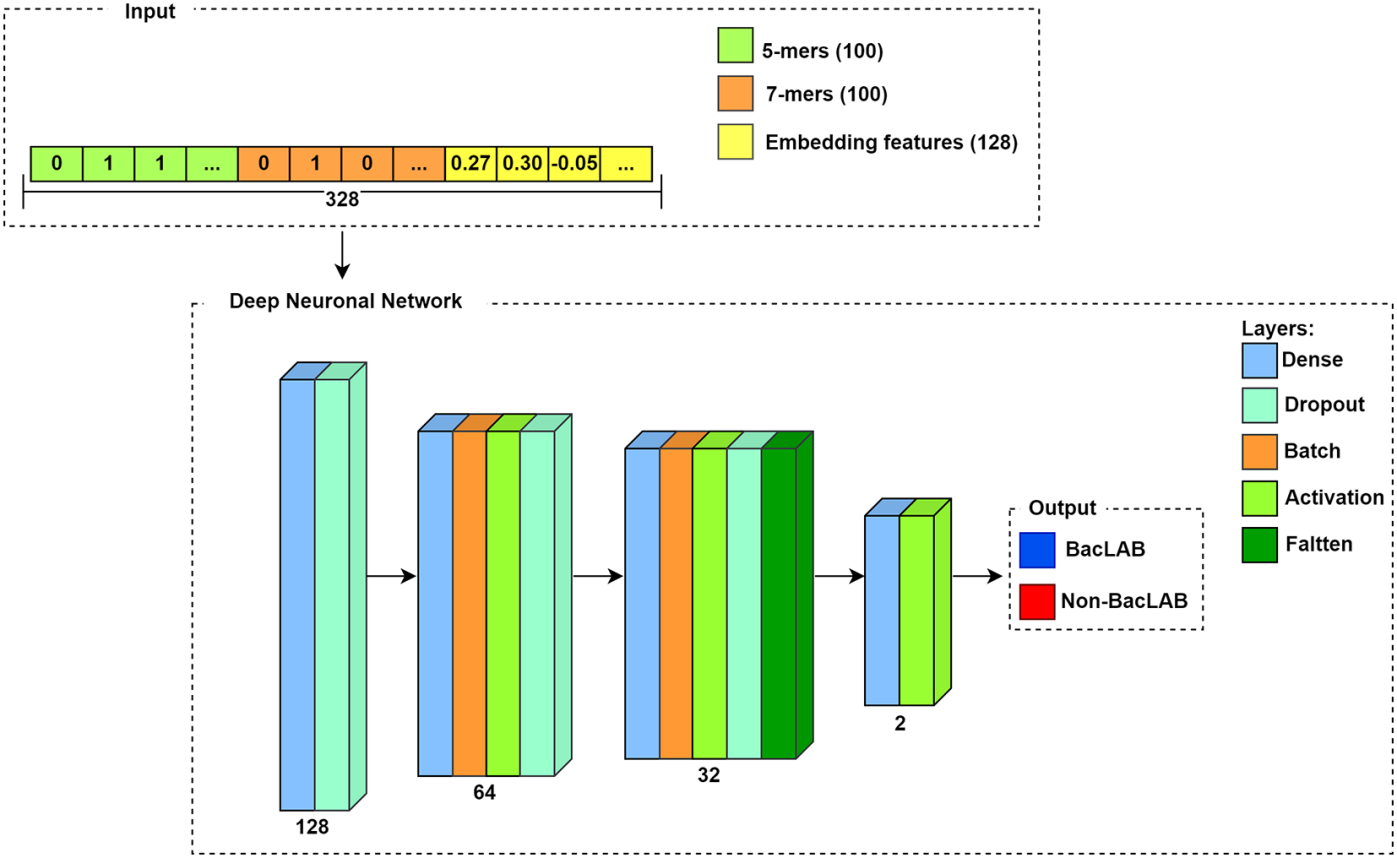
Flowchart of the deep neural network.

The network architecture consists of four blocks. The input for each sequence is a vector, which corresponds to the concatenation of the results described in the k-mers section and the embedding features. Therefore, the length of the input depends on the number of concatenated features. In Figure 7, a representation is used where the extracted results using k-mers for k=5 and k=7, and the embedding features are concatenated. Since the result in k-mers corresponds to a vector of length 100, while the embedding features provide a vector length of 128, the input corresponds to a vector length of 328 for each sequence. The output of the neural network is the class of each sequence, where 1 denotes BacLAB and 0 represents non-BacLAB.

The model established the number of neurons in each defined layer block, with 128 neurons for the first two layers, 64 neurons for the next four layers in the second block, followed by 32 neurons for the five subsequent layers in the third block, and finally, two neurons in the last two layers in the fourth block. The number of neurons was determined based on the input parameters and the DNN architecture. ^104^ Out of the total thirteen layers in the model (excluding input and output layers), four layers are dense, three layers are activation layers, three layers are dropout layers, two layers are normalization layers, and one layer is a flattening layer. Table 3 provides a summary of the layers in the proposed DNN model.

**Table 3.** Layers of the deep neural network model.

Layer	Type	Output shape	Param #
dense	Dense	(None, 128)	42112
dropout	Dropout	(None, 128)	0
dens e 1	Dense	(None, 64)	8256
batc h normalization	Batch	(None, 64)	256
activation	Activation	(None, 64)	0
dropou t 1	Dropout	(None, 64)	0
dens e 2	Dense	(None, 32)	2080
batc h normalizati on 1	Batch	(None, 32)	128
activation 1	Activation	(None, 32)	0
dropou t 2	Dropout	(None, 32)	0
flatten	Flatten	(None, 32)	0
dens e 3	Dense	(None, 2)	66
activatio n 2	Activation	(None, 2)	0

Additionally, among the hyperparameters used, 75 epochs were set, a batch size of 40, and a learning rate of 2.5×10-5 for the Adam optimizer. “Mean_absolute_error” was used as the loss function. For training and testing the neural network, the k-fold cross-validation technique was employed, with k=30 selected.

For hyperparameter tuning, an iterative approach based on cross-validation (k=30) was employed, where values ​​were progressively optimized through empirical evaluation of key metrics (loss, accuracy, and F1-score). While this method does not follow an automated search (such as grid search), it allowed for flexible adaptation to the dataset characteristics, prioritizing the balance between model stability and computational efficiency. The final hyperparameters (learning rate=2.5×10 ^−5^, batch size=40, epochs=75) were selected when consistent convergence was observed across the evaluation metrics. Given the modular nature of the model (a combination of k-mers and embeddings) and the size of the dataset, manual optimization allowed us to prioritize biologically relevant hyperparameter combinations, reducing the computational cost compared to exhaustive methods (like grid search or random search).

### Statistics analysis

In this study, ANOVA test along with Tukey test was used to assess significant differences among multiple groups based on parameters of interest, including accuracy, loss, precision, recall, and F1 score. These parameters are critical for evaluating the performance of the implemented neural network.

A confidence interval of 95% was selected to ensure that the differences identified between the groups are statistically significant, providing greater certainty about the conclusions drawn from the analysis. It is important to note that RStudio Cloud software was used as the statistical analysis tool to conduct these evaluations.

## Results

The lists of k-mers were obtained for values of k=3, k=5, k=7, k=15, and k=20. For each k-mer, the 100 most frequent repetitions among the sequences were selected. The list can be found in a xlsx file in the repository. ^105^ Through k-fold cross-validation, various performance metrics of the neural network were obtained. These metrics include loss, precision, recall, F1 score, and accuracy. They were evaluated for each group with different feature concatenations. Since thirty iterations were performed for each set, Table 4 presents the metrics averaged per group.

**Table 4.** Performance metrics obtained from k-fold cross validation (k=30) using different concatenation groups.

Group	Loss	Accuracy	Precision	Recall	F1
**EV**	10.818	89.423	0.897	0.895	0.895
**3-mers + EV**	11.500	88.648	0.889	0.887	0.887
**5-mers + EV**	10.000	90.071	0.904	0.902	0.901
**7-mers + EV**	10.100	90.049	0.903	0.901	0.901
**15-mers + EV**	10.500	89.584	0.897	0.895	0.895
**20-mers + EV**	10.300	89.763	0.899	0.898	0.898
**3-mers + 5-mers + EV**	10.900	89.184	0.893	0.891	0.891
**3-mers + 7-mers + EV**	11.600	89.085	0.893	0.892	0.892
**7-mers + 5-mers + EV**	9.900	90.143	0.903	0.901	0.901
**15-mers + 20-mers +EV**	10.200	89.885	0.900	0.899	0.899

The initial evaluation was conducted using only features extracted from EV. The results obtained for each metric demonstrate notable performance, as both precision and F1 score reached approximately 89%, while the loss function was around 10%. However, an exploration was conducted by including more features to examine if the metric percentages could be improved. Therefore, a concatenation of EV features with various k-mers was implemented.

To demonstrate if there are significant differences between the metrics of each group, an Analysis of Variance (ANOVA) was conducted for each metric. Table 5 shows the results obtained. This analysis revealed substantial differences between the groups, as the Pr(>F) values are less than α=0.05. Therefore, the null hypothesis is rejected, and the alternative hypothesis is accepted.

**Table 5.** Resultados de la prueba ANOVA. Los parámetros de la tabla indican: Df: Grados de Libertad, Sum Sq: Suma de cuadrados, Mean Sq: Cuadrado medio, Pr(>F): Valor p.

Parametro	Factor	Df	Sum Sq	Mean Sq	F value	Pr( *>*F)
**Loss**	Group	9	102.58	113.978	10.321	8.042e-14
	Residuals	290	320.27	11.044	-	-
**Acc**	Group	9	65.982	73.314	12.524	*<*2.2e-16
	Residuals	290	169.765	0.5854	-	-
**Precision**	Group	9	0.0066813	0.00074237	12.326	*<*2.2e-16
	Residuals	290	0.0174667	0.00006023	-	-
**Recall**	Group	9	0.006772	0.00075244	10.918	1.23e-14
	Residuals	290	0.019987	0.00006892	-	-
**F1 score**	Group	9	0.00656	0.00072889	10.654	2.812e-14
	Residuals	290	0.01984	0.00006841	-	-

To discern the differences between groups, a Tukey post hoc test was conducted. This test allows paired comparisons of the means of each group. Since the aim is to determine if using concatenated features yields better results than using EV exclusively. Table 6 presents the results of the Tukey test for the groups that show significant differences between using EV exclusively or the concatenation of EV with k-mers. The complete table can be found on the GitHub page.

**Table 6.** Tukey test results for accuracy comparing EV group and k-mer concatenation groups. The parameters in the table indicate: diff: difference in the means of the compared groups, lwr: lower limit of the confidence interval, upr: upper limit of the confidence interval, p adj: adjusted p-value.

Parametro	Group	Diff	lwr	Upr	p adj
**Accuracy**	**EV - 3+EV**	0.77510000	0.14519745	1.40500256	0.00424300
	**EV - 5+7+EV**	-0.72010000	-1.35000255	-0.09019745	0.01159510
	**EV - 5+EV**	-0.64796667	-1.27786922	-0.01806411	0.03805910
**Precision**	**EV - 3+EV**	7.333333E-03	0.00094401	0.01372266	0.01102510
	**EV - 5+7+EV**	-6.666667E-03	-0.01305599	-0.00027734	0.03295030
	**EV - 5+EV**	-7.333333E-03	-0.01372266	-0.00094401	0.01102510
**Loss**	**EV - 5+7+EV**	0.95456667	0.08938459	1.81974875	0.01784760
**Recall**	**EV - 3+EV**	8.333333E-03	0.00149862	0.01516804	0.00485130
**F1 score**	**EV - 3+EV**	8.333333E-03	0.00152375	0.01514292	0.00459870

In the accuracy parameter, there is a significant difference for the groups ‘3-mers + EV’, ‘5-mers + 7-mers + EV’, and ‘5-mers + EV’. These show ‘p adj’ values lower than α=0.05. The difference between the mean values of the EV group and the ‘3-mers + EV’ group in the ‘diff’ parameter yields a positive value, indicating that the results of the EV group are superior compared to ‘3-mers + EV’. Conversely, the differences of the ‘5-mers + 7-mers + EV’ and ‘5-mers + EV’ groups are negative. This indicates that using these two concatenation groups of k-mers and EV produces better accuracy results than using only EV.

For the precision parameter, the mean values of the EV group surpassed those of ‘3-mers + EV’, showing a positive difference. Similarly to accuracy, the exclusive use of EV yields superior precision. However, the groups ‘5-mers + 7-mers + EV’ and ‘5-mers + EV’ exhibited higher mean values than EV, displaying negative differences, indicating that these groups produce better precision than the exclusive use of EV. Regarding the loss parameter, significant differences were observed only between EV and the ‘EV + 5-mers + 7-mers’ group. In contrast to accuracy, the mean values of EV were higher than those of the ‘EV + 5-mers + 7-mers’ group, favoring the concatenated feature group, considering that lower loss percentages are desired in a neural network.

Results for the Recall and F1 scores showed significant differences between the EV and ‘3-mers + EV’ groups for both parameters. However, in both cases, the mean values for EV outperformed ‘3-mers + EV’. These results indicate that optimal Recall and F1 scores are generated for the EV group. The Tukey test results indicated that the ‘5-mers + 7-mers + EV’ group produces the best result. Among the cross-validation folds of this group, fold k=22 demonstrated the best result, recording a loss of 8.500%, an accuracy of 91.471%, and a precision, recall, and F1 score of 91.000%. Due to its performance, this methodology was chosen for implementation as the model’s classifier and for incorporating the weights generated in the neural network.

Notably, this accuracy surpasses or matches reported values from: General bacteriocin classifiers (e.g., 88.5% in SMO-based models, ^68^ 95.54% in SVM/RF approaches ^70^). Broader antimicrobial peptide predictors (e.g., 91.7% in AMPlify ^76^), despite addressing the more complex LAB/non-LAB distinction. While direct comparisons are limited by the absence of prior taxonomy-aware models, these benchmarks contextualize our model’s competitive edge. Full methodological comparisons are detailed in Discussion.

**Figure 8.**
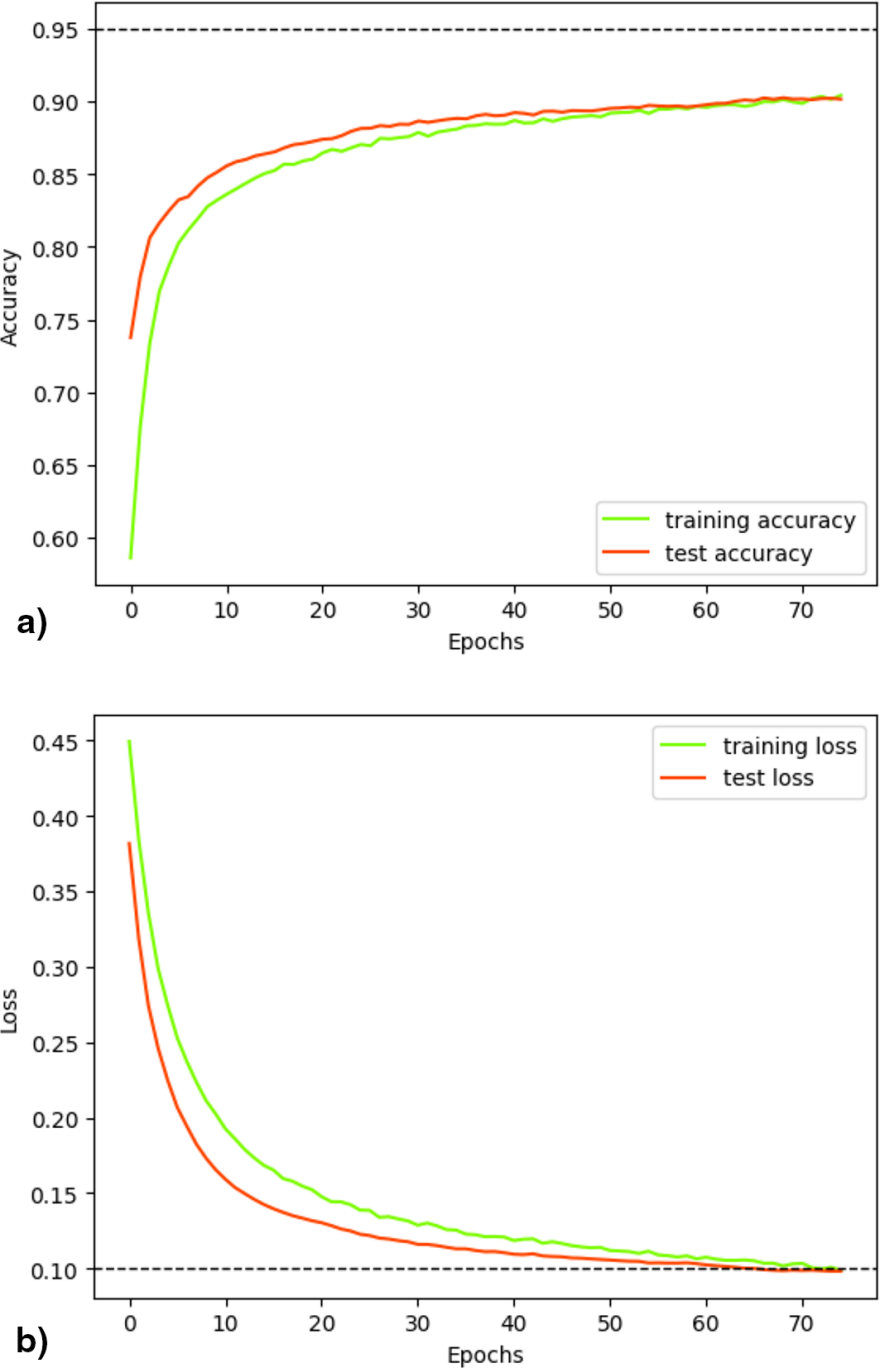
Accuracy and Loss evaluation. Visualization of the distributed training metrics for the classifier after 75 epochs from 22° folder, which yielded superior results by employing the concatenation of 5-mers, 7-mers and Embedding Vector. (a) Accuracy progression during training and validation; (b) Loss progression during training and validation.

Figure 8 illustrates the progress of the loss and accuracy metrics during the 75 epochs of fold 22. The measurements indicated adequate convergence during training. Initially, accuracy revealed low values that progressively increased over epochs, both in training and validation ( Figure 8a). In contrast, the loss was high during the initial stages of training, decreasing as the training and validation processes progressed ( Figure 8b). Although attempting to use a larger number of epochs, there was no observed increase in accuracy or decrease in loss beyond the maximum level reached at epoch 70, so this parameter was set at 75, as increasing it would imply greater computational expense without any benefit.

**Figure 9.**
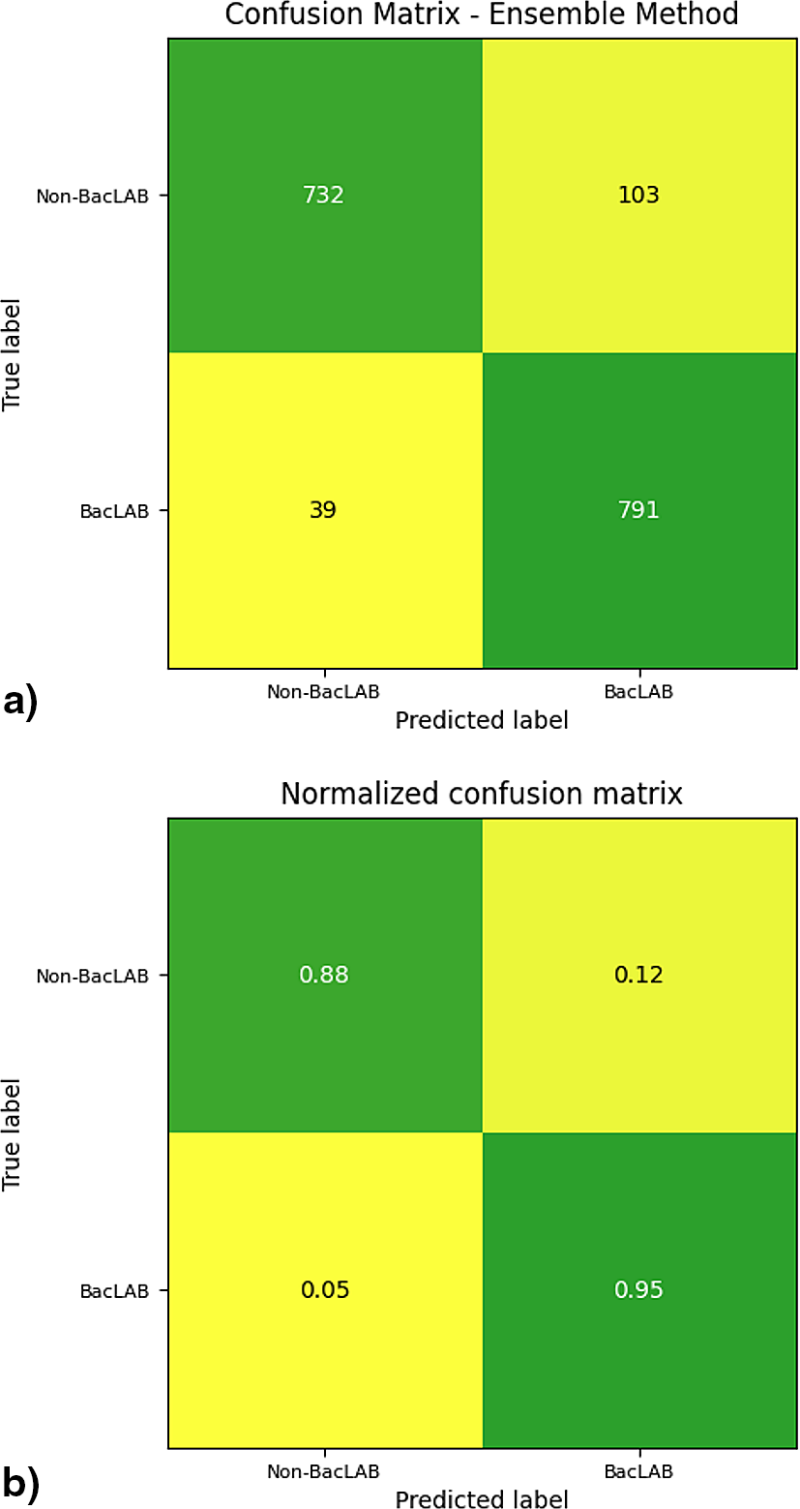
Confusion matrix of 22° folder. a) The panel shows the confusion matrix for the number of sequences evaluated. b) The panel shows the confusion matrix for the number of sequences evaluated normalized to one.

The efficiency of the neural network was assessed using a confusion matrix. The data from the main diagonal were presented, indicating the number of correct predictions made by the model ( Figure 9). A total of 732 sequences were correctly classified as non-BacLAB, while 791 were classified as true BacLAB proteins. Values below the main diagonal represent false negatives, where 39 cases were incorrectly classified as non-BacLAB. On the other hand, values above the main diagonal reflect false positives, where 103 cases were incorrectly classified as BacLAB.

## Discussion

According to the results of the Tukey test, the concatenation of EV and k-mers did not improve the evaluation metrics for all combinations. When comparing EV with ‘3-mers + EV’, decreases in metrics such as accuracy, precision, and loss were observed for the latter group. This could be caused by using a very short k-mer, which increases the probability of finding these k-mers in non-BacLAB sequences, resulting in more false positives. On the other hand, other combinations like ‘7-mers + EV’, ‘15-mers + EV’, ‘20-mers + EV’, ‘3-mers + 5-mers + EV’, ‘3-mers + 7-mers + EV’, ‘15-mers + 20-mers + EV’ did not show a statistically significant difference for any metric. And it was found that the ‘5-mers + 7-mers + EV’ group produces the best result.

The superior performance of the ‘5-mers + 7-mers + EV’ group can be attributed to the selected lengths of k-mers. In several studies, characteristic peptide sequences produced by lactic acid bacteria with lengths of 5 and 7 AA have been identified. Bacteriocins of subclass IIa contain the consensus sequence YGNGVXC at the N-terminal end that characterizes them. Similarly, sequences of leucocin A-UAL 187, sakacin P, and curvacin A had this same 7 AA pattern in their N-terminal region. ^9^^,^^97^ However, other articles consider only the highly conserved part, the first 5 AA excluding the variable AA. This characteristic sequence is YGNGV or YGNGL. ^13^^,^^106^^,^^107^ Therefore, given the precedent that certain characteristic sequences of length 5 and 7 exist among bacteriocins, this could explain why the combination of these groups yields better results.

On the other hand, the confusion matrix results showed a higher sensitivity rate than specificity. Improving specificity could be considered in future work since, for this study, higher specificity would be preferable over sensitivity. Misclassifying a non-BacLAB as BacLAB could result in losses during laboratory tests if experimental tests are to be implemented.

Regarding computational efficiency, the model was designed to balance performance and accessibility. All stages (training, validation, and testing) were run on Google Colab using free GPUs (T4/K80), demonstrating that the system does not require specialized hardware for implementation. This choice ensures that the methodology is reproducible in resource-limited academic or industrial environments, without compromising the accuracy of the results. However, while the model is viable in standard environments such as Google Colab, its performance on massive datasets (e.g. >1 million sequences) may require architectural adjustments to maintain reasonable training times.

The model developed in this study achieved results within the range reported in the literature. However, direct benchmarking against existing models is challenging, as no previous studies have specifically addressed binary classification of bacteriocins produced by LAB vs. non-LAB. For example, the BAGEL software can detect putative gene clusters of bacteriocins in new bacterial genomes and has demonstrated an ROC (Receiver Operating Characteristic) analysis value of 0.99. ^108^ Comparable to the BLASTP protein search tool, these applications use techniques to help recognize potential bacteriocin sequences by evaluating their similarity to known bacteriocins. ^109^

Similarly, there is the Bacteriocin Operon and Gene Block Associator (BOA) software, which, unlike other models, identifies homologous gene blocks associated with bacteriocins to predict new ones. ^73^ The Bacteriocin-Diversity Assessment software (v1.2 version) also performs similar operations. Although these studies mention achieving high accuracy, the specific percentage reached is not mentioned. ^110^ Additionally, a comparison was made with studies using machine learning and deep learning techniques in Table 7. In this comparison, as mentioned earlier, the study presents accuracy within the existing literature, surpassing by 3% the work done by Poorinmohammad et al. (2018) ^68^ and by 4% compared to the results obtained in Redshaw et al. (2023). ^81^

**Table 7.** Performance of our model in comparisons with other methods of machine learning.

Method	Purpose	Database	Metrics evaluated	Reference
Generation of physicochemical characteristics, support vector machine (SVM) and random forest (RF) model.	Predict bacteriocin protein sequences	283 bacteriocins and 283 non-bacteriocins	Accuracy: 95.54%	^70^
Word Embedding with Deep Recurrent Neural Networks (RNN)	Predict new bacteriocins from protein sequences without using sequence similarity.	346 bacteriocins and 346 non-bacteriocin	Accuracy: 99%	^75^
Sequential Minimal Optimization (SMO)-based classifier	Search for relevant characteristics of lantibiotics, which can be used in lantibiotic bioengineering.	280 lantibiotic and 190 non-lantibiotic	Accuracy: 88.5% Specificity: 94%	^68^
Word-embedding algorithm using biophysical properties	Design and testing of compounds derived from bacteriocins to generate 20 AA peptides that can be synthesized and their activity evaluated.	346 bacteriocins and 346 non-bacteriocins	-	^26^
Support vector machines (SVM)	Identification of biologically active and antimicrobial peptides.	2704 in total	Accuracy: 97%	^80^
Krein-support-vector machine (SVM).	Predict the overall antimicrobial activity of sequences	Two datasets: 3556 and 3246	1° Datase’s accuracy: 86-92% 2° Dataset’s accuracy: 72-77%	^79^
Embedding vectors and Deep Learning Neuronal Network (DNN) using k-mers	Identification of bacteriocins produced by LAB	24,964 BacLAB and 25,000 Non-BacLAB	Accuracy: 91.47% Loss: 8.500% Precision: 91.47 % Recall: 87.66% F1 score: 91%	This work

This work also demonstrated superior performance compared to the BACII algorithm, which identifies and classifies bacteriocin sequences. By integrating physicochemical and genomic patterns from known Class II bacteriocin families, it achieved an 86% specificity. ^34^ Similarly, a better outcome was observed compared to using sequence composition as features. In a study where this feature was used, an accuracy of 90.55% was achieved. ^84^ Although a similar result was observed compared to the work of Dua et al. (2020), which achieved an accuracy of 91.7%. ^111^ However, it’s important to consider that each study uses varying amounts of data for their respective articles.

Although the model has demonstrated strong performance in its results, it is important to consider that the sequence filtering step (50 ≤ length ≤ 2000 amino acids), while ensuring a manageable range for training, introduces two main limitations. First, there is a length bias in k-mer representation. Longer sequences naturally contain more subfragments (k-mers), which increases the likelihood of matching characteristic k-mers from the feature list—even if those matches are not biologically relevant. This can lead to a higher chance of false positives in longer sequences, potentially compromising the accuracy of the classification.

Second, standardizing variable-length sequences into fixed-size k-mer vectors (100 features) results in the loss of structural information that depends on the original sequence length. While k-mers are effective at capturing local motifs, they do not preserve information about the relative position of those motifs within the full sequence. As a result, important structural patterns, such as domain arrangements in distant regions, may be lost during the vectorization process.

On the other hand, our model was trained using the best-characterized LAB genera (Lactobacillus, Enterococcus, etc.), which are the most abundant in public databases. For example, in UniProt (the database used in this study), 62% of LAB bacteriocin sequences correspond to Lactobacillus, while genera such as Weissella represent only 3.5%. Although we employed stratified cross-validation to reduce bias, this disparity could affect the detection of atypical bacteriocins in rare genera. Future studies could enrich the dataset with experimental isolates from underrepresented taxa.

Future iterations of the model could address these limitations by incorporating normalization weights based on sequence length to correct for bias, or by including positional k-mers—such as dividing the sequence into segments and extracting k-mers from each region independently. Additionally, the validation was limited to computational data. While the model demonstrated high precision (91.47%), its real-world applicability would require in vitro experimental testing to confirm whether the sequences classified as BacLAB actually produce functional bacteriocins. Furthermore, it is necessary to verify whether the identified k-mers are truly associated with antimicrobial activity. These experiments, although crucial, fall outside the scope of this study and represent a valuable direction for future research.

## Conclusion

In this study, we developed a deep learning neural network for binary classification of bacteriocin sequences, successfully distinguishing LAB-produced bacteriocins from non-LAB sequences. Our approach combining k-mer features (k=3,5,7,15,20) and embedding vectors achieved optimal performance with the ‘5-mers+7-mers+EV’ configuration, demonstrating 91.47% accuracy and 8.50% loss in the best fold (k=22). These results compare favorably with existing bacteriocin classification tools, outperforming some by 3-10%, despite addressing the more challenging LAB/non-LAB distinction.

Key strengths of our approach include the identification of 500 characteristic k-mers that may serve as signatures for LAB bacteriocins. Also, validation on a large, balanced dataset (≈25,000 sequences per class), and computational efficiency via Google Colab implementation

However, we acknowledge important limitations, like taxonomic bias. Public databases overrepresent certain LAB genera (e.g., Lactobacillus), potentially affecting model generalizability to rare producers. In the same way, we have some problems with sequence length constraints. Our 50-2000 AA filter may exclude structurally important extremes. And, the lack of experimental validation. Predicted bacteriocins require in vitro confirmation of biological activity.

Future work could: expand taxonomic diversity through targeted sequencing of underrepresented LAB, investigate k-mer positional conservation within full-length sequences, and validate top predictions through antimicrobial assays. These advances would strengthen the model’s utility for developing targeted antimicrobials in food safety and therapeutic applications.

### Ethics and consent

Ethical approval and consent were not required.

## Data Availability

Zenodo: Deep Learning Neural Network Development for the Classification of Bacteriocin Sequences Produced by Lactic Acid Bacteria: Repository. https://doi.org/10.5281/zenodo.13279718. ^105^ This project contains the following underlying data: **Software-Related Files**:
• BacLABNet_script.ipynb (Deep Learning Neural Network for classification of Bacteriocin Sequences) • embed_proteins.py (Recurrent Neural Network to obtained the embedding vectors) • model_I22.h5 (This file contains the trained weights of the trained model) • model_I22.json (This file contains the structure of the trained model) • rnn_gru.pt (Initial weights of the Recurrent Neural Network to obtain embedding vectors) • List_kmers.csv (List of 5-mers and 7-mers obtained from dataset after it filtered sequences shorter than 50 aa and longer than 2000 aa) **Files Used for Training, Testing, and Validation of the Neural Network**
• data_nonBacLAB.csv (25000 nonBacLAB amino acid sequences retrieved from Uniprot) • data_BacLAB.csv (24964 BacLAB amino acid sequences retrieved from Uniprot) Data are available under the terms of the Creative Commons Zero “No rights reserved” data waiver (CC0 1.0 Public domain dedication). Zenodo: Deep Learning Neural Network Development for the Classification of Bacteriocin Sequences Produced by Lactic Acid Bacteria: Repository. https://doi.org/10.5281/zenodo.13279718. ^105^
•
data_BacLAB_and_nonBacLAB.csv (Combination of sequences from data_BacLAB.csv and data_nonBacLAB.csv) • all k.mers list.xlsx (Table of all k-mers obtained for k=3,5,7,15,20) Data are available under the terms of the ( Creative Commons Zero “No rights reserved” data waiver (CC0 1.0 Public domain dedication).

## Software availability

Source code available from: https://github.com/lady1004/BacLAB-Deep-Learning-Neural-Network.

Archived source code at time of publication: https://doi.org/10.5281/zenodo.13279718.

License: CC0 1.0 Universal.
