# Supplementary material for: Deep learning neural network development for the classification of bacteriocin sequences produced by lactic acid bacteria
Source: F1000Res. 2025 Jun 20;13:981. Originally published 2024 Aug 30. [Version 2] doi: 10.12688/f1000research.154432.2 (PMC12332477; doi:10.12688/f1000research.154432.2)
Supplement: Peer review history

# Reviewer report: Reviewer response for version 2

**DOI:** 10.5256/f1000research.183776.r393480

**Published:** 2025-08-07

**Authors:** John J. Georrge

**Affiliations:** 1 University of North Bengal, Darjeeling, India

The authors revised the manuscript as per the reviewers comments. The article may be indexed.

Is the work clearly and accurately presented and does it cite the current literature?

Yes

If applicable, is the statistical analysis and its interpretation appropriate?

Yes

Are all the source data underlying the results available to ensure full reproducibility?

Yes

Is the study design appropriate and is the work technically sound?

Yes

Are the conclusions drawn adequately supported by the results?

Yes

Are sufficient details of methods and analysis provided to allow replication by others?

Yes

Reviewer Expertise:

Rational Drug Designing, Novel Drug Target Identification, Cheminformatics, Bioinformatics, Molecular Modelling, Docking, QSAR, Pharmacohpore, Protein Engineering, Bioactive Peptides, Reverse Vaccinology, Artificial Intelligence, and Machine Learning.

I confirm that I have read this submission and believe that I have an appropriate level of expertise to confirm that it is of an acceptable scientific standard.

# Reviewer report: Reviewer response for version 2

**DOI:** 10.5256/f1000research.183776.r393916

**Published:** 2025-08-07

**Authors:** Cristóbal Joel González-Pérez

**Affiliations:** 1 Centre for Food Research and Development, Hermosillo, Sonora, Mexico

In this research, a deep learning neural network was developed for bacteriocin sequence classification, which distinguishes bacteriocins produced by LAB from those produced by non-LAB. I consider this relevant because currently, we lack tools to support these bioinformatics searches.

 Suggestions: 

 TABLE 1. In examples of Class II, change Pediocina for Pediocin A or Pediocin.

 TABLE 1. In examples of Class IV, eliminate one “e” in Eenterocin.

 In Fields where bacteriocins can be applied to address diverse issues, in the subtitle Medicine, it is important to mention that no bacteriocin is currently being used due to the long process that must be gone through; they are important promising molecules but none have been accepted for application in humans.

 In page 5, in the last parragraph, check “BACII?”, change or check “?” for “α”.

 In the statistical analysis, the authors must mention whether the data are normal or not, that is, if they performed normality tests, they must be mentioned, since Tukey's is for parametric data.

 In the second paragraph, page 19, “in vitro” must be italic.

Is the work clearly and accurately presented and does it cite the current literature?

Yes

If applicable, is the statistical analysis and its interpretation appropriate?

I cannot comment. A qualified statistician is required.

Are all the source data underlying the results available to ensure full reproducibility?

Yes

Is the study design appropriate and is the work technically sound?

Yes

Are the conclusions drawn adequately supported by the results?

Yes

Are sufficient details of methods and analysis provided to allow replication by others?

Yes

Reviewer Expertise:

Microbiology (Bacteriocins)

I confirm that I have read this submission and believe that I have an appropriate level of expertise to confirm that it is of an acceptable scientific standard.

# Reviewer report: Reviewer response for version 2

**DOI:** 10.5256/f1000research.183776.r393911

**Published:** 2025-08-07

**Authors:** Ismail Erol

**Affiliations:** 1 Bahcesehir University, Istanbul, Turkey

After revision, the authors substantially increased the quality of the manuscript. I have no futher comments. The current status of the manuscript is ready for indexing. 

 Ensuring the reproducibility of research is really important, and the authors considered that and shared their code via Github. 

 It is acknowledged that the authors have considered reproducibility.

Is the work clearly and accurately presented and does it cite the current literature?

Yes

If applicable, is the statistical analysis and its interpretation appropriate?

Yes

Are all the source data underlying the results available to ensure full reproducibility?

Yes

Is the study design appropriate and is the work technically sound?

Yes

Are the conclusions drawn adequately supported by the results?

Yes

Are sufficient details of methods and analysis provided to allow replication by others?

Yes

Reviewer Expertise:

Molecular Modeling

I confirm that I have read this submission and believe that I have an appropriate level of expertise to confirm that it is of an acceptable scientific standard.

# Reviewer report: Reviewer response for version 2

**DOI:** 10.5256/f1000research.183776.r393481

**Published:** 2025-07-03

**Authors:** Alaa Kareem Niamah

**Affiliations:** 1 University of Basrah, Basrah, Basra Governorate, Iraq

After a second review of the manuscript, I see that the authors have made the necessary corrections. I believe the manuscript is now clearer and ready for indexing.

Is the work clearly and accurately presented and does it cite the current literature?

Partly

If applicable, is the statistical analysis and its interpretation appropriate?

Yes

Are all the source data underlying the results available to ensure full reproducibility?

Yes

Is the study design appropriate and is the work technically sound?

Yes

Are the conclusions drawn adequately supported by the results?

Partly

Are sufficient details of methods and analysis provided to allow replication by others?

Partly

Reviewer Expertise:

Bacteriocin production ,  Lactic acid Bacteria

I confirm that I have read this submission and believe that I have an appropriate level of expertise to confirm that it is of an acceptable scientific standard.

# Reviewer report: Reviewer response for version 1

**DOI:** 10.5256/f1000research.169463.r355998

**Published:** 2025-01-27

**Authors:** Alaa Kareem Niamah

**Affiliations:** 1 University of Basrah, Basrah, Basra Governorate, Iraq

Dear Editors and Authors 

 1-The introduction needs to be supported by some sources related to the current study, such as: refer 1 and 2

 ‏

 2-The aim of the study is not clear and is not mentioned in the manuscript introduction.

 3-The labeling of the x-axis in Figure 2 should be corrected.

 4-Table 2 has no meaning and should be deleted. Since the coding is known and previously included in references.

 5-Figure 6 is not clear. It should be explained better than the current situation and explain what these shapes, such as circles and arrows, mean.

 6-Table 8 should preferably be written and discussed with the results of previous references, not as in the current situation. It is not permissible to put a table containing the results of others.

 7-Figure 8 The authors did not explain what A and B mean.

 8-Figure 8 The authors did not explain what a and b mean.

 9-The conclusions are very poor. This chapter is dedicated to the conclusions of the current study, but we see that the authors have mentioned many results. This chapter should be rewritten and all the results mentioned should be deleted.

Is the work clearly and accurately presented and does it cite the current literature?

Partly

If applicable, is the statistical analysis and its interpretation appropriate?

Yes

Are all the source data underlying the results available to ensure full reproducibility?

Yes

Is the study design appropriate and is the work technically sound?

Yes

Are the conclusions drawn adequately supported by the results?

Partly

Are sufficient details of methods and analysis provided to allow replication by others?

Partly

Reviewer Expertise:

Bacteriocin production ,  Lactic acid Bacteria

I confirm that I have read this submission and believe that I have an appropriate level of expertise to confirm that it is of an acceptable scientific standard, however I have significant reservations, as outlined above.

## References

[1] : Structure, mode of action and application of pediocin natural antimicrobial food preservative: A review. *Basrah Journal of Agricultural Sciences* .2018;31(1) : 10.37077/25200860.2018.76 59-69 10.37077/25200860.2018.76

[2] : Emerging lactic acid bacteria bacteriocins as anti-cancer and anti-tumor agents for human health. *Heliyon* .2024;10(17) : 10.1016/j.heliyon.2024.e37054 e37054 10.1016/j.heliyon.2024.e37054 39286220 PMC11402949

# Reviewer report: Reviewer response for version 1

**DOI:** 10.5256/f1000research.169463.r355994

**Published:** 2025-01-11

**Authors:** John J. Georrge

**Affiliations:** 1 University of North Bengal, Darjeeling, India

**Abstract**

 The research investigates a deep learning neural network for the binary classification of bacteriocin sequences produced by lactic acid bacteria (LAB). Utilizing k-mers and vector embeddings for feature extraction, the study tested ten group combinations, concluding that the concatenated features of 5-mers, 7-mers, and embedding vectors (EV) yielded superior results. With k-fold cross-validation (k=30), the model achieved notable accuracy (91.47%), precision, recall, and F1 score (91%). These results demonstrate a promising approach for identifying bacteriocins, paving the way for applications in medicine, livestock, aquaculture, and food preservation.

**Weaknesses:** The abstract lacks explicit mention of the practical challenges or limitations, such as computational expense or data imbalance, which are critical for assessing the feasibility of real-world applications.

**Introduction**

 The study highlights the growing challenge of antibiotic resistance and the potential of bacteriocins as alternatives. LAB bacteriocins, recognized as GRAS (Generally Recognized as Safe) and QPS (Qualified Presumption of Safety), show promise for therapeutic and industrial applications. The introduction emphasizes the critical need for efficient classification methods, leveraging artificial intelligence (AI) and deep learning to address the limitations of traditional genomic tools. Existing methods like BAGEL and BLASTP rely heavily on sequence homology, often leading to false negatives. The study proposes a deep neural network as an innovative solution for identifying LAB-produced bacteriocins.

**Weaknesses:** While the introduction effectively frames the problem, it does not provide sufficient detail on the limitations of existing deep learning models, nor does it address the potential biases introduced by selecting specific LAB genera.

**Methods**

 The research employed amino acid (AA) sequences sourced from the UniProt database, filtered for lengths between 50 and 2000 AAs. LAB bacteriocin sequences were labelled as “BacLAB,” and non-LAB sequences as “Non-BacLAB.” Feature extraction was performed using k-mers of varying lengths (3, 5, 7, 15, and 20) and embedding vectors generated via a Gated Recurrent Unit (GRU)-based recurrent neural network (RNN). The concatenated features were inputs to a deep neural network (DNN) structured in four blocks with 13 layers. The model used Adam optimization, a mean absolute error loss function, and k-fold cross-validation (k=30). Statistical analyses included ANOVA and Tukey tests to validate performance metrics.

**Weaknesses:** The methods section lacks clarity on how hyperparameters were tuned and does not justify the selection of specific k-mer lengths.

**Results**

 The study identified five lists of 100 characteristic k-mers for each selected length. Performance metrics from cross-validation demonstrated that the concatenation of 5-mers, 7-mers, and embedding vectors (5-mers+7-mers+EV) achieved the best results with:

- Loss: 8.50%
- Accuracy: 91.47%
- Precision, Recall, F1 Score: 91.00%

Significant differences in accuracy and loss were observed between groups. The confusion matrix revealed high sensitivity but lower specificity, highlighting potential areas for improvement. Compared to other machine learning models, this study’s approach exceeded accuracy benchmarks by 3-10%.

**Weaknesses:** While the results are promising, the limited scope of comparison with other algorithms leaves room for a more robust benchmarking process.

**Discussion**

 The superior performance of the 5-mers+7-mers+EV group is attributed to characteristic motifs in bacteriocins. The study’s findings align with known sequences in subclass IIa bacteriocins, such as YGNGVXC. The results underscore the importance of selecting appropriate k-mer lengths to balance specificity and sensitivity. However, limitations include the model’s higher false positive rate for non-BacLAB sequences, indicating room for refinement in feature selection. Additionally, comparisons with existing tools like BAGEL reveal the unique advantage of the proposed approach in distinguishing LAB-specific bacteriocins.

**Weaknesses:** The discussion does not critically evaluate the computational cost and scalability of the model for larger datasets. It also overlooks the potential need for experimental validation of the predictions to confirm biological relevance.

**Conclusion**

 The study successfully developed a deep learning-based classification model for LAB bacteriocins, achieving consistent results comparable to and sometimes exceeding existing methods. The identified k-mers and embeddings offer a robust foundation for future work in therapeutic, aquacultural, and industrial applications. While promising, further specificity and practical testing improvements are essential to validate and expand the model’s utility.

**Weaknesses:** The conclusion is overly optimistic and does not sufficiently address the study’s limitations, such as the reliance on publicly available datasets that may not represent all bacteriocin-producing LAB.

**References**

 References were cited appropriately within the research, highlighting the breadth of prior work on bacteriocins and machine learning applications.

Is the work clearly and accurately presented and does it cite the current literature?

Yes

If applicable, is the statistical analysis and its interpretation appropriate?

Yes

Are all the source data underlying the results available to ensure full reproducibility?

Yes

Is the study design appropriate and is the work technically sound?

Yes

Are the conclusions drawn adequately supported by the results?

Yes

Are sufficient details of methods and analysis provided to allow replication by others?

Yes

Reviewer Expertise:

Rational Drug Designing, Novel Drug Target Identification, Cheminformatics, Bioinformatics, Molecular Modelling, Docking, QSAR, Pharmacohpore, Protein Engineering, Bioactive Peptides, Reverse Vaccinology, Artificial Intelligence, and Machine Learning.

I confirm that I have read this submission and believe that I have an appropriate level of expertise to confirm that it is of an acceptable scientific standard, however I have significant reservations, as outlined above.
